# Addressing the Gaps in Mortality Data: A Case for National Mortality Surveillance

**DOI:** 10.4269/ajtmh.23-0042

**Published:** 2023-04-10

**Authors:** Ties Boerma

**Affiliations:** Institute for Global Public Health, Community Health Science, University of Manitoba, Winnepeg, Manitoba, Canada

Population-based data on mortality by age, sex, and cause of death, further disaggregated by location and multiple other dimensions of inequality, are the statistical foundation of public health. Yet, such information is lacking in almost all high-mortality countries. In the absence of well-functioning birth and death registration systems, countries rely on cross-sectional household surveys to partially fill the gap on all-cause mortality levels and on special studies with medical certification or verbal autopsy to obtain some information on medical causes of death. These two sources are still a far cry from the required population-based mortality information for effective programs.

Global production of detailed cause-specific mortality estimates for countries has increased dramatically, using increasing complex statistical models, but that is not a substitute for sound country cause of death data, as is availability in low-mortality countries. Sadly, the availability of reliable empirical country data on mortality by cause has only made piecemeal progress. The *AJTMH* supplement presents the results of a major effort in Mozambique to fill this data gap by establishing a national mortality surveillance system.

Was it possible to generate meaningful mortality data? The project established a national sample of 700 geographic clusters with about 740,000 people under prospective surveillance in the 11 provinces of a vast resource-constrained country. Verbal autopsies provided a comprehensive picture of the cause-specific mortality fractions for major age groups during 2019–2020.^1^ Social autopsies generated further information on health seeking behaviors during the severe conditions preceding death.^2^ Population mortality rates were presented for national and provincial levels and considered plausible when compared with other sources and global estimates.

Was it affordable? The annual operating costs for the Mozambique system were about US$1 million, or US$1–1.3 per person enrolled.^3^ That translates into US$0.03–0.04 per capita per year for the whole population. This would be only slightly higher than the average annual per capita costs of a national demographic and health survey system with one survey every 5 years. International programs, notably the Demographic and Health, and Multiple Indicators Cluster Surveys, have provided sustained support for household survey programs in many low- and lower-middle income countries. It is long overdue to expand this strategy to sample-based vital statistics systems.

Can the all-cause mortality statistics be taken at face value? The results of the project’s own assessment of completeness of birth and death reporting through a parallel household survey were sobering: about half of the vital events were missed.^4^ This, however, is no showstopper because adjustments for underreporting can be made. It does show the need for independent parallel data collection on a subsample of population under surveillance to assess the magnitude of biases and to continually take measures to enhance data quality. A dual system (surveillance and survey) is the foundation of the sample registration system in India, which has been generating mortality and fertility data by state for decades. The project results also suggest that special community cadres for data collection and reporting are a requirement and that simply adding reporting births and death information to the menu of community health workers is not a good option.

Can the data on the cause of death patterns be trusted? Verbal autopsy is an imperfect tool. Minimally invasive tissue sampling (MITS) is a technological advance and a socially acceptable procedure, as has been shown for deceased children in Mozambique^5^ and elsewhere, that can greatly improve the cause of death ascertainment. The Mozambique work shows how information on cause-specific mortality from the sample vital statistics system and a MITS research study can improve national statistics on causes of death by reducing misclassification and considering multiple causes of death.^6^^,^^7^

Was it time to “transition” the project? The Mozambique government’s aim was to generate a national system for mortality rates and causes of death for all ages. The motivation of the main donor, the Bill & Melinda Gates Foundation, was slightly different. Its interest was primarily to learn how autopsy data, using MITS, could improve causes of death in children under 5 years of age. This perhaps explains why the donor asked for a “transition plan” to local institutions halfway through the project life span and ended its funding after 4 years. The challenges of such a rapid transition of an ambitious, large-scale project in a low-resource (in terms of financing and specialist staff) country are well described in this supplement.^8^

If we are serious about improving the fundamentals of public health, a concerted and sustained effort is needed from global donors, international agencies, and country governments to address the painful gap in information on mortality by cause in high-mortality settings. Efforts to improve civil registration and vital statistics in all countries are commendable and necessary but will unlikely solve the mortality statistics gap in the near future. Mortality surveillance systems with verbal autopsy,^9^ combined with population-based surveys and improved medical certification of the cause of death and reporting by health facilities, are a critical intermediate step toward full birth and death (with cause) registration systems and need to be prioritized. The ability to measure excess mortality due to the COVID-19 pandemic on a regular basis is just one example of the immediate value of such systems for public health.^10^
